# Developmental Profiles of Young Deaf and Hard of Hearing Children and Their Associated Predictors

**DOI:** 10.1111/cch.70129

**Published:** 2025-07-03

**Authors:** Natalie Zehnwirth, Libby Smith, Daisy A. Shepherd, Valerie Sung

**Affiliations:** ^1^ The Royal Children's Hospital Parkville Victoria Australia; ^2^ Monash Children's Hospital Clayton Victoria Australia; ^3^ Murdoch Children's Research Institute Parkville Victoria Australia; ^4^ The University of Melbourne Parkville Victoria Australia

**Keywords:** child development, deaf, early intervention

## Abstract

**Background:**

Concomitant developmental disability is common in deaf and hard of hearing (DHH) children. We describe the early developmental profiles of DHH children and explore factors that may be predictive of these profiles.

**Methods:**

We report on data from DHH children aged 0–66 months who are participants of a longitudinal child hearing databank in Victoria, Australia. Developmental profiles were measured using the Ages and Stages Questionnaire (ASQ) across five domains (communication, gross motor, fine motor, personal social and problem solving). We reported descriptive statistics and used logistic regression to estimate odds ratios and determined which characteristics were associated with below cut‐off ASQ scores.

**Results:**

Caregivers of 882 children aged 0–66 months completed the ASQ between 2012 and 2022. A considerable proportion of children scored below their developmental expectations for age with 35% below ASQ cut‐off for communication, 24% fine motor, 23% problem solving, 21% gross motor and 20% personal social. Children with a mild degree of hearing loss scored below cut‐off ranging from 16% to 26% across the domains. Predictive factors for below cut‐off development included admission to neonatal intensive care, extreme prematurity, infection requiring intravenous antibiotics and having more than one comorbidity for all domains. Bilateral hearing loss, cochlear implant use, jaundice requiring treatment and seizures were associated with communication delays. Cochlear implant use was a protective factor for gross motor development.

**Conclusions:**

Early developmental screening is vital for DHH children, as these children have multiple developmental needs. Degree of hearing loss does not predict overall development; however, children with a mild degree of hearing loss can have developmental impairments and benefit from developmental monitoring. Early targeted intervention to support DHH children is imperative in maximizing their functional abilities and well‐being.

**Summary:**

- Deaf and hard of hearing children may have additional developmental disabilities and comorbidities, and early intervention supports their development.
- This paper provides insights into the specific factors that influence development in young deaf and hard of hearing children drawn from a large sample of universally early‐identified children.
- All deaf and hard of hearing children should receive developmental monitoring, including children with mild hearing loss. Developmental monitoring should target deaf and hard of hearing children who were born premature, those who received early medical interventions at birth and those with medical comorbidities.

## 1 Introduction

Hearing loss is a common congenital condition, affecting 1–3 in every 1000 children (Korver et al. 2017). Each year, the Universal Newborn Hearing Screening (UNHS) identifies approximately 600 Australian infants with congenital hearing loss (Sung, Smith, et al. 2019). Early identification of clinically significant hearing loss is crucial, as the child may need additional support to enhance their development. A diagnosis of hearing loss is usually accompanied by a search for an underlying aetiology and may be associated with co‐existing developmental disability and medical comorbidities. Approximately one‐third of children who are deaf and hard of hearing (DHH) have an additional developmental disability (Corrales and Oghalai 2013), which may be explained by a combination of the aetiology of the hearing loss and any associated risk factors. Despite early diagnosis and early intervention of hearing loss, language outcomes of DHH children are on average a full standard deviation below population means (Sung, Smith, et al. 2019).

Communication and language outcomes in DHH children are well documented in the literature; however, motor and social development in these children has received comparatively less attention, with limited literature describing the developmental profiles of young DHH children. Hearing loss can affect skill acquisition in other developmental areas, particularly when the aetiology for the child's hearing loss has vestibular, neurological or multi‐system involvement. Children who are DHH often demonstrate suboptimal motor skills, compared to their typically hearing peers (Vidranski and Farkaš 2015; Gheysen et al. 2007). Established factors influencing improved developmental outcomes of DHH children include earlier identification of hearing loss, earlier enrolment in early intervention services, the absence of additional disability and higher level of maternal education (Ching et al. 2013; Ching et al. 2018; Yoshinaga‐Itano 2003). One study suggests that children with hearing loss diagnosed before 6 months of age have better motor, social and communication skills than those diagnosed between 6 and 12 months and 12–18 months, due to the benefits of early amplification and early intervention therapies (Sahli 2019).

Early identification of co‐existing disability in children with hearing loss allows families to engage with early intervention services and has the potential to alter their developmental trajectory. Ages and Stages Questionnaire (ASQ) validation studies from US Centers for Disease Control and Prevention estimate a prevalence of one developmental disability in 12%–16% of the typical population (Squires et al. 2009). Evidence suggests that the support provided by early intervention services for families and their children alters long‐term development and reduces psychosocial and secondary health complications (The Royal Australasian College of Physicians 2013). Caregiver questionnaires can serve as an invaluable tool in the early stages of identifying children who may require further developmental assessment. The ASQ (Squires and Bricker 2009) is a widely used and extensively evaluated screening tool that can be completed by caregivers to assess a child's functional abilities. The ASQ is now in its 3rd edition (ASQ‐3) and consists of 21 age‐specific questionnaires that relate to five primary areas of development: communication, gross motor, fine motor, problem solving and personal social (Squires and Bricker 2009). The tool has been translated into numerous languages and evaluated across diverse cultural communities throughout the world (Singh et al. 2017). The ASQ has acceptable sensitivity and specificity rates for use as a screening tool (86%–87% and 85%–95%, respectively), assesses children aged from 2 to 66 months and takes approximately 15 minutes for a caregiver to complete (Singh et al. 2017; Vitrikas et al. 2017; Oberklaid and Drever 2011).

The purpose of this study is to describe the early developmental profiles of DHH children as measured by the ASQ and to explore the individual child and family characteristics that are predictive of these profiles.

## 2 Methods

This was a cross‐sectional study of Australian children enrolled in the Victorian Childhood Hearing Longitudinal Databank (VicCHILD). VicCHILD is a state‐wide population‐based longitudinal databank that commenced enrolment in 2012 and is open to every child with permanent hearing loss in the state of Victoria (population around 6.6 million and annual birth rate approaching 74 000) (Sung, Smith, et al. 2019; Australian Bureau of Statistics 2021a; Australian Bureau of Statistics 2022). The majority of children enrolled in VicCHILD are identified and approached through the Victorian Infant Hearing Screening Program (VIHSP), which offers a hearing screen to 99.5% of Victorian babies in the days or weeks after birth and follows them to definitive hearing diagnosis (Sung, Smith, et al. 2019). Details of VicCHILD recruitment methodology are described elsewhere (Sung, Smith, et al. 2019). VicCHILD has ethics approval from the Royal Children's Hospital Human Research and Ethics Committee (approval number 31081), with parent/caregivers providing informed written consent.

At enrolment, families complete a custom‐designed questionnaire containing baseline demographic information and an age‐appropriate ASQ. ASQ scores in the domains of communication, gross motor, fine motor, personal social and problem solving were recorded. A score in the below cut‐off range falls less than two standard deviations below the mean, indicating the child is functioning below developmental expectations for their age (Squires et al. 2009). Severity of hearing loss is classified as mild (21–40 dB), moderate (41–60 dB), severe (61‐90 dB) or profound (> 91 dB). Laterality of hearing loss is noted and classified as unilateral or bilateral. For children with asymmetric bilateral hearing loss, the severity is graded according to the better hearing side. The parent and child demographic and clinical predictors explored were maternal and paternal level of education, primary language spoken at home, socio‐economic status (measured using the Socio‐Economic Indexes for Areas [‘SEIFA’]), fever during pregnancy, sex, gestational age, birthweight, degree, laterality and type of hearing loss, device use, comorbidities (defined as the presence of an additional medical condition co‐occurring with hearing loss), enrolment in early intervention, bacterial meningitis, infection requiring intravenous antibiotics, jaundice requiring phototherapy or exchange transfusion, seizures and admission to neonatal intensive care (NICU). The SEIFA disadvantage index ranks postcodes across Australia using census data from the Australian Bureau of Statistics. It reflects the socio‐economic conditions of people in an area based on their access to resources and ability to participate in society (Australian Bureau of Statistics 2021b). The index takes into account factors like average household education, income, employment status and disability for each postcode (Australian Bureau of Statistics 2021b). A score of 1000 is the state‐wide average, with lower scores indicating more disadvantaged areas and higher scores indicating more advantaged areas (Australian Bureau of Statistics 2021b).

Demographic and clinical characteristics of participants were summarized, reporting the mean and standard deviation for continuous variables and proportions for categorical variables. Responders were compared to non‐responders to understand how representative the sample was of the target population and whether there was potential selection bias. Univariable logistic regression was used to describe the associations between predictive factors individually and the outcome of below cut‐off ASQ scores for each of the five domains. Calculation of odds ratios (OR) with 95% confidence intervals (CI) was estimated for each predictor of interest and the outcome variable (below cut‐off development score) separately. Subgroup analysis by sex was conducted to explore whether the univariable predictors differed by child sex. We used Stata program Version 17 for the statistical analysis using available cases only, with any missing data quantified and reported where appropriate (StataCorp 2021).

## 3 Results

**FIGURE 1:**
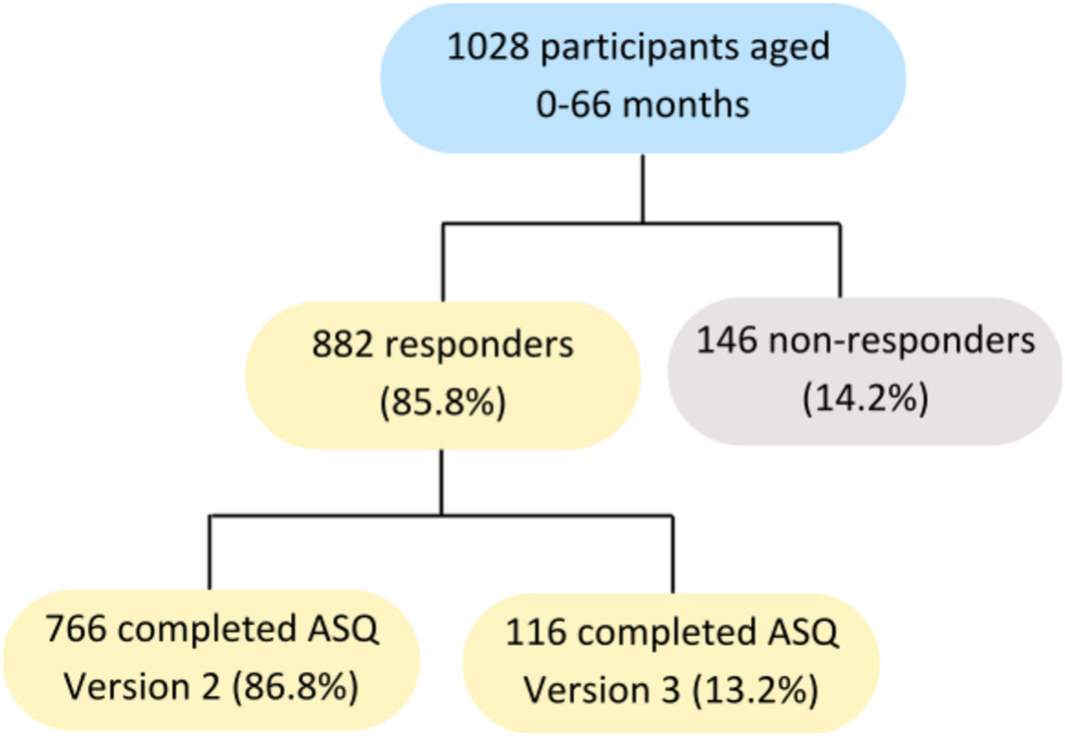
Flowchart of participants.

A total of 1028 children aged 0–66 months were recruited between 2012 and January 2022. Of those recruited, 882 (85.8%) participant parents completed the baseline questionnaire, 766 (86.8%) completed ASQ Version 2, and 116 (13.2%) completed ASQ Version 3 (Figure 1).

### 3.1 Study Participants

The participants' demographic and clinical characteristics are presented in Table 1. Responders lived in areas of less socio‐economic disadvantage (higher SEIFA scores) than non‐responders; otherwise, responder and non‐responder characteristics were similar. In the study sample, there were slightly more males than females. The mean age of participant children at the time of recruitment was 15.3 months (SD 14.6 months), and the mean age at hearing loss diagnosis was 1.7 months (SD 2.9 months). The majority of participant children had sensorineural hearing loss (73.8%), and just over two‐thirds had bilateral hearing loss (67.7%). The degree of hearing loss was spread relatively evenly. The majority of participant children did not use a device (56.4%) at the time of enrolment, and 52% of participant children had one or more comorbidities.

**TABLE 1:** Demographic and child characteristics of responders and non‐responders.

	Responders	Non‐responders
	*n* = 882	*n* = 146
	*n* (%)	*n* (%)
Child characteristics		
Sex: male	479 (54.3)	62 (45.3)
Age at recruitment in months: mean (SD)	15.3 (14.6)	17.2 (18.0)
Age at diagnosis in months: mean (SD)	1.7 (2.9)	
Missing data	135 (15.3)	
*Gestational age in weeks: mean (SD)*	38.2 (3.2)	38.4 (2.6)
• Extreme preterm (< 28 weeks)	21 (2.4)	2 (1.4)
• Very preterm (28–32 weeks)	12 (1.4)	1 (0.7)
• Moderate to late preterm (32–37 weeks)	82 (9.3)	13 (8.9)
• Not preterm (> 37 weeks)	767 (87.0)	130 (89.0)
Missing data	18 (2.0)	0 (0)
*Birthweight in kilograms* ^*a*^ *: mean (SD)*	3.2 (0.7)	3.1 (0.6)
Missing data	436 (49.4)	
Degree of hearing loss^b^		
• Mild	217 (24.6)	28 (19.2)
• Moderate	243 (27.5)	46 (31.5)
• Severe	155 (17.6)	24 (16.4)
• Profound	192 (21.8)	25 (17.1)
• Unknown	75 (8.5)	23 (15.8)
*Hearing laterality* ^*c*^		
• Bilateral hearing loss	597 (67.7)	81 (55.5)
• Unilateral hearing loss	277 (31.4)	56 (38.4)
• Unknown	8 (0.9)	9 (6.2)
*Type of hearing loss*		
• Sensorineural hearing loss	651 (73.8)	91 (62.3)
• Auditory neuropathy	78 (8.8)	10 (6.9)
• Mixed	81 (9.2)	9 (6.2)
• Conductive	22 (2.5)	8 (5.5)
• Atresia	31 (3.5)	13 (8.9)
Missing data	19 (2.2)	15 (10.3)
*Device use*		
• Hearing aid	294 (33.3)	41 (28.1)
• Cochlear implant	91 (10.3)	10 (6.9)
• No device	497 (56.4)	95 (65.1)
*Presence of comorbidities* ^*a*^		
• None	201 (22.8)	14 (9.6)
• 1 or more	220 (24.9)	11 (6.8)
Missing data	461 (52.2)	121 (82.9)
*Enrolment in early intervention* ^*a*^		
• Yes/referred	428 (48.5)	59 (40.4)
• No	159 (18.0)	21 (14.4)
Missing data	295 (33.5)	66 (45.2)
**Family characteristics**		
*Maternal education level*		
• Not complete	123 (13.9)	10 (6.8)
• Year 12	249 (28.2)	25 (17.1)
• Tertiary	418 (47.4)	52 (35.6)
Missing data	92 (10.4)	59 (40.4)
*Paternal education level*		
• Not complete	168 (19.0)	18 (12.3)
• Year 12	255 (28.9)	19 (13.0)
• Tertiary	306 (34.7)	44 (30.1)
Missing data	153 (17.3)	65 (44.5)
*Education level of parent completing questionnaire*		
• Not complete	120 (13.6)	9 (6.2)
• Year 12	248 (28.1)	25 (17.1)
• Tertiary	431 (48.9)	54 (37.0)
Missing data	83 (9.4)	58 (39.7)
*Primary language spoken at home*		
• English	628 (71.2)	68 (46.6)
• Other including Auslan	223 (25.3)	24 (16.4)
Missing data	31 (3.5)	54 (37.0)
*SEIFA disadvantage index*: mean (SD)	1007.4 (63.8)	996.4 (58.2)
**Antepartum risk factor** ^**a**^		
Fever during pregnancy		
• No	402 (45.6)	72 (49.3)
• Yes	42 (4.8)	4 (2.7)
Missing data	438 (49.7)	70 (47.9)
**Postpartum risk factors** ^**a**^		
*Bacterial meningitis*		
• No	429 (48.6)	70 (47.9)
• Not sure	13 (1.5)	2 (1.4)
• Yes	5 (0.6)	0 (0)
Missing data	438 (49.7)	74 (50.7)
*Infection requiring intravenous antibiotics*		
• No	368 (42.0)	66 (45.2)
• Not sure	17 (1.9)	2 (1.4)
• Yes	59 (6.7)	4 (2.7)
Missing data	438 (49.7)	74 (50.7)
*Jaundice requiring exchange transfusion or phototherapy*		
• No	397 (45.0)	67 (45.9)
• Not sure	14 (1.6)	1 (0.7)
• Yes	35 (4.0)	4 (2.7)
Missing data	436 (49.4)	74 (50.7)
*Seizures*		
• No	420 (47.6)	1 (48.6)
• Not sure	10 (1.1)	0 (0)
• Yes	14 (1.6)	1 (0.7)
Missing data	438 (49.7)	74 (50.7)
*Admission to neonatal intensive care unit*		
• No	692 (78.5)	93 (63.7)
• Yes	159 (18.0)	25 (17.1)
Missing data	31 (3.5)	28 (19.2)

^a^ Birthweight, comorbidity, enrollment in early intervention, antepartum and postpartum risk factor data collected from 2016 onwards.

^b^ In better ear if bilateral, or affected ear if unilateral.

^c^ ‘Unknown’ includes participants with auditory neuropathy and atresia.

**FIGURE 2:**
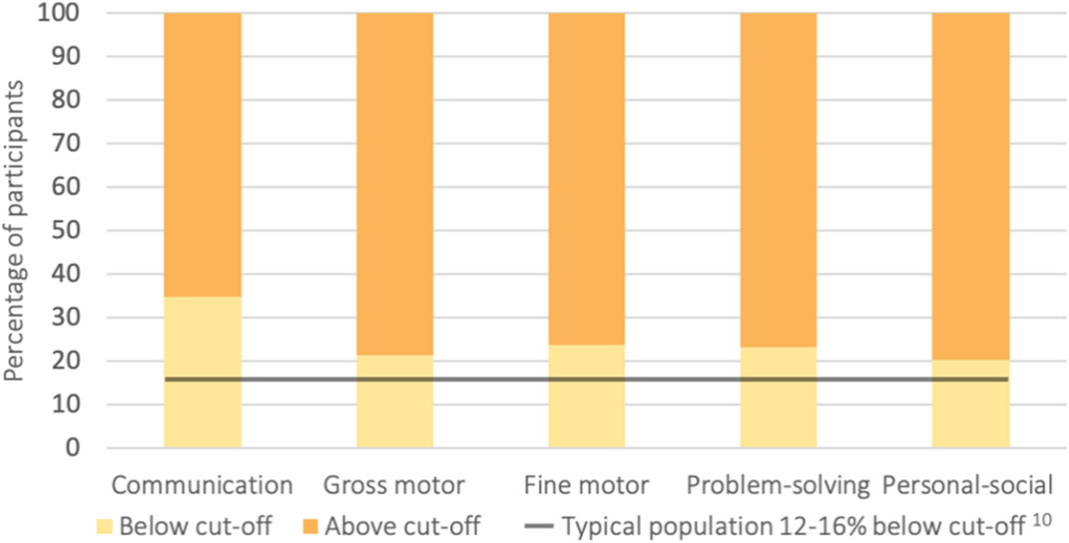
Overall ASQ domain cut‐off scores.

**FIGURE 3:**
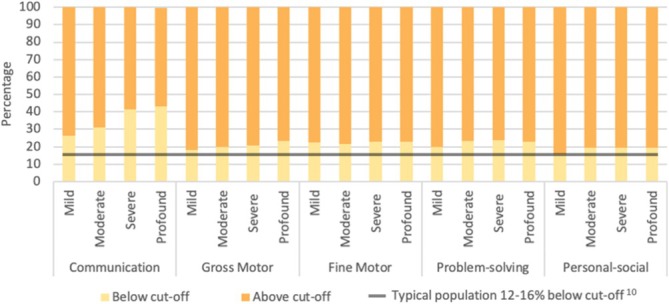
Degree of hearing loss and developmental cut‐off score.

Figure 2 shows the overall proportion of children (*n* = 882) with below cut‐off scores for each developmental domain: 35% for communication, 24% for fine motor, 23% for problem solving, 21% for gross motor and 20% for personal social. Figure 3 shows the overall proportion of children with below cut‐off scores for each developmental domain, broken down by degree of hearing loss. Proportion estimates (alongside 95% CI) for domain cut‐off scores grouped by degree of hearing loss are shown in Table 2. The association between individual predictive factors and below cut‐off ASQ domain scores are shown in Table 3 and stratified by sex in Table 4.

**TABLE 2:** Proportion estimates for domain cut‐off scores grouped by degree of hearing loss.

	Communication	Gross motor	Fine motor	Problem solving	Personal social
	Proportion [95% CI]	Proportion [95% CI]	Proportion [95% CI]	Proportion [95% CI]	Proportion [95% CI]
Mild hearing loss					
Below cut‐off	0.26 [0.21–0.33]	0.18 [0.14–0.24]	0.22 [0.17–0.29]	0.20 [0.15–0.26]	0.16 [0.12–0.21]
Above cut‐off	0.73 [0.67–0.79]	0.81 [0.76–0.86]	0.77 [0.71–0.83]	0.80 [0.74–0.85]	0.84 [0.78–0.88]
Moderate hearing loss					
Below cut‐off	0.31 [0.25–0.37]	0.20 [0.15–0.25]	0.22 [0.17–0.27]	0.23 [0.18–0.29]	0.19 [0.15–0.25]
Above cut‐off	0.69 [0.63–0.75]	0.80 [0.74–0.85]	0.78 [0.73–0.83]	0.77 [0.71–0.82]	0.80 [0.75–0.85]
Severe hearing loss					
Below cut‐off	0.41 [0.33–0.50]	0.21 [0.15–0.28]	0.23 [0.17–0.31]	0.24 [0.17–0.31]	0.19 [0.13–0.27]
Above cut‐off	0.55 [0.50–0.66]	0.79 [0.72–0.85]	0.77 [0.69–0.83]	0.76 [0.69–0.83]	0.81 [0.73–0.86]
Profound hearing loss					
Below cut‐off	0.43 [0.36–0.51]	0.23 [0.18–0.30]	0.23 [0.17–0.30]	0.23 [0.17–0.30]	0.19 [0.14–0.26]
Above cut‐off	0.57 [0.49–0.64]	0.77 [0.70–0.82]	0.77 [0.70–0.83]	0.77 [0.70–0.83]	0.80 [0.74–0.86]

Abbreviation: CI, confidence interval.

**TABLE 3:** Estimated odds ratios for each individual clinical and demographic predictor and below cut‐off domain score.

	Communication	Gross motor	Fine motor	Problem solving	Personal social
	OR [95% CI]	OR [95% CI]	OR [95% CI]	OR [95% CI]	OR [95% CI]
Demographic variables					
Sex					
• Male versus female	1.3 [1.0–1.7]	1.1 [0.8–1.5]	1.7 [1.2–2.3]	1.3 [1.0–1.8]	1.3 [0.9–1.8]
Age at diagnosis (months)	0.9 [0.9–1.0]	0.9 [0.9–1.0]	0.9 [0.9–1.0]	1.0 [0.9–1.0]	1.0 [0.9–1.0]
Primary language spoken at home					
• English versus other	1.0 [0.7–1.4]	0.9 [0.7–1.4]	1.0 [0.7–1.4]	0.9 [0.6–1.3]	1.0 [0.7–1.5]
Education level of parent completing questionnaire					
• Not complete versus year 12 or higher	1.0 [0.8–1.2]	1.1 [0.9–1.4]	1.3 [1.1–1.6]	1.3 [1.1–1.6]	1.0 [0.8–1.3]
Primary language spoken at home					
• English versus other	1.0 [0.7–1.4]	0.9 [0.7–1.4]	1.0 [0.7–1.4]	0.9 [0.6–1.3]	1.0 [0.7–1.5]
SEIFA disadvantage index	1.0 [1.0–1.0]	1.0 [1.0–1.0]	1.0 [1.0–1.0]	1.0 [1.0–1.0]	1.0 [1.0–1.0]
Clinical variables					
Gestation					
• Extreme prematurity versus not extreme prematurity	1.8 [1.4–2.3]	1.7 [1.4–2.3]	1.7 [1.3–2.3]	1.7 [1.3–2.3]	1.8 [1.4–2.3]
Birthweight (kilograms)	1.7 [1.3–2.4]	1.9 [1.4–2.7]	1.5 [1.1–2.1]	1.7 [1.2–2.3]	1.9 [1.3–2.6]
Degree of hearing loss					
• Moderate versus mild	1.3 [0.8–1.8]	1.1 [0.7–1.8]	1.0 [0.6–1.5]	1.2 [0.8–2.0]	1.3 [0.8–2.1]
• Severe versus mild	2.0 [1.3–3.1]	1.2 [0.7–2.0]	1.0 [0.6–1.7]	1.3 [0.7–2.1]	1.3 [0.7–2.2]
• Profound versus mild	2.2 [1.4–3.3]	1.4 [0.8–2.2]	1.0 [0.6–1.6]	1.2 [0.7–2.0]	1.3 [0.8–2.1]
Hearing laterality					
• Bilateral versus unilateral	2.2 [1.6–3.0]	1.2 [0.8–1.7]	1.1 [0.8–1.6]	1.3 [0.9–1.9]	1.5 [1.0–2.2]
Device use					
• Hearing aid versus no device	1.1 [0.8–1.4]	0.9 [0.6–1.3]	1.0 [0.7–1.4]	1.1 [0.8–1.5]	1.1 [0.8–1.6]
• Cochlear implant versus no device	1.8 [1.2–2.9]	0.5 [0.3–1.0]	0.7 [0.4–1.2]	0.7 [0.4–1.2]	0.9 [0.5–1.7]
Comorbidities					
• >/= 1 versus none	1.9 [1.3–2.8]	2.1 [1.3–3.6]	2.0 [1.2–3.3]	1.8 [1.1–3.0]	2.2 [1.3–3.8]
Fever during pregnancy					
• Yes versus no	0.9 [0.4–1.8]	0.9 [0.4–2.0]	0.8 [0.4–1.8]	1.7 [0.9–3.4]	0.9 [0.4–2.1]
Bacterial meningitis					
• Yes versus no	3.5 [0.6–20.9]	2.7 [0.5–16.6]	5.3 [0.9–32.0]	2.5 [0.4–15.0]	2.7 [0.5–16.7]
Infection requiring intravenous antibiotics					
• Yes versus no	2.3 [1.3–4.0]	3.2 [1.8–5.8]	2.8 [1.5–5.0]	2.6 [1.4–4.7]	2.4 [1.3–4.4]
Jaundice requiring exchange transfusion or phototherapy					
• Yes versus no	2.4 [1.2–4.8]	1.6 [0.7–3.4]	1.8 [0.9–3.8]	2.6 [1.3–5.4]	2.5 [1.2–5.3]
Seizures					
• Yes versus no	4.4 [1.4–13.2]	5.4 [1.8–16.0]	1.9 [0.6–5.9]	1.5 [0.5–4.9]	1.6 [0.5–5.3]
Admission to neonatal intensive care					
• Yes versus no	2.0 [1.4–2.8]	2.7 [1.8–3.9]	2.4 [1.6–3.4]	2.0 [1.4–2.9]	2.8 [1.9–4.1]
Enrolment in early intervention services					
• Enrolled/referred versus not enrolled	3.0 [1.9–4.8]	1.2 [0.7–1.8]	1.3 [0.8–2.1]	2.1 [1.3–3.4]	2.0 [1.2–3.2]

Abbreviations: CI, confidence interval; OR, odds ratio.

**TABLE 4:** Sex‐stratified estimated odds ratios of predictor variables and below cut‐off domain score.

	Communication OR [95% CI]	Gross motor OR [95% CI]	Fine motor OR [95% CI]	Problem‐solving OR [95% CI]	Personal social OR [95% CI]
Birthweight					
• Male	1.5 [1.0–2.3]	1.7 [1.1–2.6]	1.6 [1.1–2.4]	1.7 [1.1–2.6]	2.0 [1.2–3.0]
• Female	2.1 [1.3–3.4]	2.3 [1.4–3.7]	1.6 [0.9–2.7]	1.1 [1.1–1.2]	1.8 [1.1–3.0]
Prematurity					
• Male	2.0 [1.4–2.9]	1.7 [1.2–2.4]	1.6 [1.2–2.3]	1.8 [1.3–2.5]	1.9 [1.3–2.7]
• Female	1.6 [1.1–2.3]	1.7 [1.2–2.5]	1.6 [1.2–2.3]	1.6 [1.1–2.3]	1.7 [1.2–2.5]
Gestation					
• Male	1.1 [1.1–1.2]	1.1 [1.0–1.2]	1.1 [1.0–1.2]	1.1 [1.0–1.2]	1.1 [1.1–1.2]
• Female	1.1 [1.0–1.2]	1.1 [1.0–1.2]	1.1 [1.0–1.2]	1.1 [1.0–1.2]	1.1 [1.0–1.2]
Comorbidities					
• Male	0.6 [0.5–0.8]	0.6 [0.5–0.8]	0.7 [0.5–0.8]	0.6 [0.5–0.8]	0.6 [0.5–0.8]
• Female	0.8 [0.6–1.0]	0.6 [0.4–0.8]	0.5 [0.4–0.7]	0.6 [0.4–0.8]	0.5 [0.4–0.7]
Early intervention					
• Male	0.4 [0.2–0.6]	0.8 [0.5–1.3]	1.0 [0.6–1.5]	0.6 [0.4–1.0]	0.6 [0.3–0.9]
• Female	0.4 [0.3–0.7]	0.6 [0.3–1.0]	0.3 [0.2–0.6]	0.3 [0.2–0.6]	0.7 [0.4–0.7]
Fever in pregnancy					
• Male	1.5 [0.5–3.9]	0.5 [0.2–1.4]	0.9 [0.4–2.4]	0.5 [0.2–1.2]	0.8 [0.3–2.2]
• Female	0.8 [0.3–2.3]	5.3 [0.7–41.3]	3.4 [0.4–26.8]	0.8 [0.6–2.5]	1.9 [0.4–8.5]
Bacterial meningitis					
• Male	0.5 [0.1–1.9]	0.8 [0.2–3.3]	0.3 [0.1–1.2]	0.2 [0.0–1.0]	0.3 [0.1–1.3]
• Female	0.6 [0.2–1.4]	0.4 [0.2–1.1]	0.4 [0.2–1.0]	0.7 [0.3–1.8]	0.6 [0.2–1.4]
Infection requiring IV antibiotics					
• Male	0.6 [0.4–0.8]	0.6 [0.4–0.9]	0.6 [0.4–0.9]	0.6 [0.3–0.9]	0.9 [0.6–1.3]
• Female	0.7 [0.5–1.1]	0.4 [0.2–0.6]	0.4 [0.3–0.7]	0.5 [0.3–0.8]	0.3 [0.2–0.5]
Jaundice requiring treatment					
• Male	0.6 [0.4–1.0]	0.8 [0.5–1.2]	0.7 [0.5–1.1]	0.6 [0.4–1.0]	0.7 [0.5–1.1]
• Female	0.6 [0.3–1.1]	0.8 [0.4–1.5]	1.0 [0.4–2.4]	0.6 [0.3–1.1]	0.5 [0.3–0.9]
Seizures					
• Male	0.2 [0.1–0.6]	0.4 [0.2–0.9]	0.7 [0.3–1.4]	0.7 [0.3–1.4]	0.8 [0.4–1.8]
• Female	0.7 [0.4–1.5]	0.4 [0.2–0.9]	0.8 [0.3–1.7]	0.8 [0.3–1.7]	0.6 [0.3–1.2]
Admission to NICU					
• Male	0.5 [0.3–0.8]	0.4 [0.2–0.6]	0.4 [0.3–0.7]	0.5 [0.3–0.8]	0.4 [0.2–0.6]
• Female	0.5 [0.3–0.9]	0.4 [0.2–0.7]	0.5 [0.2–0.9]	0.5 [0.3–1.0]	0.3 [0.2–0.6]

### 3.2 Communication

For the communication domain, the odds of scoring below‐cut off were 1.8 times higher for children who were born extremely premature compared to those who were not extremely premature (OR 1.8, 95% CI [1.4–2.3]), had a cochlear implant compared to no device (OR 1.8, 95% CI [1.2–2.9]), or had greater than 1 comorbidity compared to no comorbidity (OR 1.9, 95% CI [1.3–2.8]). Children with severe (OR 2.0, 95% CI [1.3–3.1]) and profound hearing loss (OR 2.2, 95% CI [1.4–3.3]) had higher odds of scoring below cut‐off than those with mild hearing loss. Odds were also higher for children with bilateral hearing loss than unilateral (OR 2.2, 95% CI [1.6–3.0]). Odds were approximately two times higher for children who had an infection that required intravenous antibiotics (than no antibiotics) (OR 2.3, 95% CI [1.3–4.0]), jaundice requiring exchange transfusion or phototherapy (than no jaundice) (OR 2.4, 95% CI [1.2–4.8]) and admission to NICU (than no admission) (OR 2.0, 95% CI [1.4–2.8]). Children who had seizures (compared to no seizures) had 4 times higher odds of scoring below cut‐off for communication (OR 4.4, 95% CI [1.4–13.2]).

### 3.3 Fine and Gross Motor

For fine motor development, the odds of scoring below cut‐off were 1.7 times higher for males (OR 1.7, 95% CI [1.2–2.3]) and extremely premature children (OR 1.7, 95% CI [1.3–2.3]). The odds of scoring below cut‐off in fine motor skills were 2 times higher for children who had more than 1 comorbidity (OR 2.0, 95% CI [1.2–3.3]), were admitted to NICU (OR 2.4, 95% CI [1.6–3.4]) or required intravenous antibiotics (OR 2.8, 95% CI [1.5–5.0]). For children with bacterial meningitis, their odds of scoring below cut‐off were 5 times higher (OR 5.3, 95% CI [0.9–32.0]) than those without.

The odds of having below average gross motor skills was lower for those with a cochlear implant compared to those with no device (OR 0.5, 95% CI [0.3–1.0]).

### 3.4 Problem‐Solving

Factors associated with below cut‐off scores for problem‐solving were extreme prematurity (OR 1.7 95% CI [1.3–2.2]), and a child having more than 1 comorbidity (OR 1.8, 95% CI [1.1–3.0]). The odds of scoring below cut‐off in problem‐solving were 2 times higher for children who had an infection requiring intravenous antibiotics (OR 2.6, 95% CI [1.4–4.7]), jaundice requiring treatment (OR 2.6, 95% CI [1.3–5.4]) and admitted to NICU (OR 2.0, 95% CI [1.4–2.9]) compared to those who did not require treatment, or admission to NICU.

### 3.5 Post‐Partum Risk Factors

Children who were born extremely premature compared to those who were not had 1.8 times greater odds of scoring below the cut‐off in their personal‐social skills (OR 1.8, 95% CI [1.4–2.3]). The odds for children who had greater than one comorbidity (OR 2.2, 95% CI [1.3–3.8]), an infection requiring intravenous antibiotics (OR 2.4, 95% CI [1.3–4.4]), jaundice requiring treatment (OR 2.5, 95% CI [1.2–5.3]) and admission to NICU (OR 2.8, 95% CI [1.9–4.1]) were over two times higher in scoring below the cut‐off in personal‐social development when compared to children who did not require treatment or admission.

We found that for every 1kilogram decrease in birthweight, the odds of DHH children scoring below cut‐off were at least 1.7 times higher in the domains of communication, gross motor, problem‐solving and personal‐social skills. There was a stronger effect observed in females with decreasing birthweight in the communication domain with the odds of scoring below‐cut off 2.1 times higher (OR 2.1 95% CI [1.3–3.4]) compared to 1.5 times in males (OR 1.5 95% CI [1.1–2.6]). A similar pattern was observed in the gross motor domain, with odds 2.3 times higher in females (OR 2.3 95% CI [1.4–3.7]), compared to 1.7 times in males (OR 1.7 95% CI [1.1–2.6]). The odds of scoring below cut‐off across all domains were between 2.5 and 5.3 times greater for children who had bacterial meningitis compared to those who did not.

For children enrolled in or referred to early intervention services, the odds of having below‐average communication skills were 3 times higher (OR 3.0 95% CI [1.9–4.8]), below‐average problem‐solving abilities 2.1 times higher (OR 2.1 95% CI [1.3–3.4]) and below‐average personal‐social skills 2 times higher (OR 2.0 95% CI [1.2–3.2]) compared to those not enrolled in these services.

Some predictors, such as fever during pregnancy or seizures, had wide confidence intervals (especially among females) reflecting the lack of precision due to small subgroup sample sizes.

## 4 Discussion

### 4.1 Statement of Principal Findings

This study is the largest of its kind to report on the developmental profiles of young DHH children at a population level in Australia. Our study supports previous data showing a higher predominance of developmental disability in DHH children, with at least 1 in 5 children demonstrating below cut‐off scores in their development, as compared to 12%–16% in normative population studies. Our study showed a strong association between below cut‐off development scores on screening assessment with the presence of comorbidities, birthweight and variables related to admission to NICU such as extreme prematurity, jaundice requiring exchange transfusion or phototherapy and infection requiring intravenous antibiotics.

### 4.2 Strengths and Weaknesses

A strength of our study is the state‐wide population approach, large sample size and high response rate of participants. Our results are generalizable to Australian children with all severities and types of hearing loss identified via UNHS and clinical services. The primary limitation of our study is that the ASQ has not been formally validated for use in the DHH population. Modification of the ASQ for screening in DHH children has been investigated by Wiley and colleagues and has been shown to adequately detect communication and gross motor deficits but has poorer positive predictive value for detecting deficits in fine motor, cognitive and personal‐social areas (Wiley and Meinzen‐Derr 2013). There is no ‘one size fits all’ screening tool, and further research is needed to develop a validated screening tool for the accurate assessment of development in DHH children.

Some respondents did not complete all survey questions, explaining the smaller number of responses in some categories. From 2016, caregivers also reported on birthweight, comorbidity, enrolment in early intervention, antepartum and postpartum data. As the VicCHILD Databank is an opt‐in process, there may be selection bias towards families from advantaged socio‐economic backgrounds or those who are attuned to recognizing developmental differences in their children.

Our study could further be strengthened by interrogating the comorbidities and hearing loss aetiologies within the DHH population to gain a deeper understanding of how these may independently affect a child's development. In addition, longitudinal analysis of our cohort could investigate how these early developmental profiles result in longer term developmental trajectories and educational outcomes.

### 4.3 Findings in Relation to Other Studies

Extreme prematurity was associated with adverse developmental profiles, consistent with established evidence on the neurological consequences of decreasing gestational age and birthweight. Previous literature indicates almost half of infants born between 22 and 24 weeks' gestation will have some form of neurodevelopmental impairment (Ream and Lehwald 2018). Both lower gestational age and reduced birthweight have been shown to independently increase the risk of such impairments, even when controlling for factors like hearing loss (Ream and Lehwald 2018). Our finding of an association between seizures and communication deficits has also been observed in unselected premature infants (Ream and Lehwald 2018). Overall, low birthweight and prematurity emerged as consistent risk factors for adverse developmental profiles across both sexes, with a stronger impact observed among females in communication and gross motor domains for decreasing birthweight. Existing literature has found that preterm males are more disadvantaged in gross motor impairment compared to females (Christensen et al. 2025).

The presence of one or more comorbidities was a clinically significant factor associated with below cut‐off development across all domains. The presence of medical comorbidities has been reported to be as high as 69% for children with bilateral hearing loss and 60% for unilateral hearing loss, with positive correlations found between the number of comorbidities and the number of health services utilized (Olivier et al. 2022).

Increasing degrees of severity of hearing loss predicted below cut‐off communication scores; this finding is consistent with previous literature (Wake et al. 2016). However, degree of hearing loss did not necessarily predict overall development; children with mild hearing loss also experienced developmental profile scores lower than expected than normative scores. Previous findings regarding early intervention and communication outcomes show an association with improved abilities (Yoshinaga‐Itano 2003; Moeller 2000). Our study found that children were more likely to score below‐cut off in communication, problem‐solving and personal‐social skills when enrolled or referred to early intervention. This likely reflects their lower developmental functioning at the time of referral, prior to receiving the full benefits of the intervention. These findings underscore the importance of early intervention support, and highlight the need for ongoing monitoring to assess developmental progress over time.

Literature regarding motor skills within the DHH population largely focuses on school‐aged children (Rajendran et al. 2012) and often demonstrates that these children are delayed in motor skills when compared with hearing peers (Gheysen et al. 2007). The finding of an association between improved scores on gross motor development in children with cochlear implants compared to no device is surprising. An explanation of this finding is challenging as comparisons are difficult to elicit from current literature as studies are small and primarily focus on older children. Anecdotally, gross motor delay is common in cochlear‐implanted children but also in DHH children without a device. Some children with cochlear implants show variable motor abilities such as delayed walking compared to their hearing‐impaired peers without cochlear implants (De Kegel et al. 2015). This may be reflective of the aetiology of the child's hearing loss, a more severe degree of hearing loss and possible alterations to vestibular function during implantation (De Kegel et al. 2015). The evidence on the effect of cochlear implantation on motor development in children with coexisting developmental disability is inconsistent and likely related to the use of different measures used to examine motor abilities in children. Worse early gross motor skills may be related to the inherent vestibular problems that arise as a result of the hearing loss aetiology and/or comorbidities of the child, for example, Usher or Pendred syndromes (De Kegel et al. 2015). Although we did not observe an association between gross motor skills and degree of hearing loss, there was an association found with the number of comorbidities, suggesting that the underlying aetiology is an important factor. Considering the diversity of reported motor skills in the DHH population, and in particular young or cochlear‐implanted children, there is a need to further examine the intrinsic motor abilities in this population and the associated predictors of the described variability.

Although some studies have reported an association between higher level of maternal education and improved language outcomes (Ching et al. 2018), this was not the case in our study. This could suggest that DHH children in our population may have appropriate access to health services irrespective of socio‐economic status. In addition, we did not see an association between the level of education and social and motor skill development of DHH children unlike previous research (Leigh et al. 2015).

### 4.4 Study Implications

Our study supports the Australasian consensus guidelines (Sung, Downie, et al. 2019) that all DHH children should be referred to a paediatrician for developmental monitoring, with the data from our study highlighting important additional risk factors associated with developmental impairment. Our study also highlights the developmental needs of children with mild hearing loss, a group who may not be eligible for early intervention services or may not be well supported by current services and health systems. This has important implications to funding models and further highlights the need to support all children with hearing loss, regardless of hearing loss severity.

## 5 Conclusion

Young DHH children have multiple developmental needs, in particular children who were born premature or required medical interventions at birth, and those with other medical comorbidities. Although the degree of hearing loss does not predict overall development, children with a mild hearing loss may also experience developmental impairments. This has important practical implications as children with mild hearing loss detected by universal newborn hearing screening may still benefit from developmental monitoring. Our findings support the need for adequate developmental surveillance and early intervention services for all DHH children regardless of hearing loss severity. Developmental monitoring should especially target DHH children who were born premature, those who received early medical interventions at birth and those with medical comorbidities.

## Author Contributions

**Natalie Zehnwirth:** writing – original draft, writing – review and editing, conceptualization, data curation, formal analysis, visualization, methodology, project administration. **Libby Smith:** methodology, data curation, supervision, writing – review and editing, conceptualization, formal analysis, project administration. **Daisy A. Shepherd:** writing – review and editing, formal analysis, supervision, conceptualization, methodology. **Valerie Sung:** conceptualization, writing – review and editing, supervision, methodology, project administration.

## Data Availability

The data that support the findings of this study are available on request from the corresponding author. The data are not publicly available due to privacy or ethical restrictions.
